# Supplementary material for: Divergent Effects of Glycemic Control and Bariatric Surgery on Circulating Concentrations of TMAO in Newly Diagnosed T2D Patients and Morbidly Obese
Source: Diagnostics (Basel). 2022 Nov 14;12(11):2783. doi: 10.3390/diagnostics12112783 (PMC9689652; doi:10.3390/diagnostics12112783)
Supplement: Supplementary file 1 [file diagnostics-12-02783-s001.zip › diagnostics-1959583-supplementary.pdf]

diagnostics

MDPI

**Table S1.** SRM transitions and collision energies used in LC-MS/MS for the detection of TMAO, $\gamma$BB and d3-MeCar(IS)

| Compound | Transition | Collision energy |
|---|---|---|
| TMAO | 76 → 58 | 16 |
| TMAO | 76 → 59 | 8 |
| $\gamma$BB | 146 → 87 | 16 |
| $\gamma$BB | 146 → 60 | 12 |
| d3-MeCar (IS) | 165 → 63 | 16 |
| d3-MeCar (IS) | 165 → 103 | 16 |
